# Postępująca Rodzinna Cholestaza Wewnątrzwątrobowa Typu 3

**DOI:** 10.34763/devperiodmed.20182204.385389

**Published:** 2019-01-14

**Authors:** Patryk Lipiński, Irena Jankowska

**Affiliations:** 1 Klinika Gastroenterologii, Hepatologii, Zaburzeń Odżywiania i Pediatrii, Instytut „Pomnik – Centrum Zdrowia Dziecka”, Warszawa, Polska

**Keywords:** cholestaza, postępująca rodzinna cholestaza wewnątrzwątrobowa, gen *ABCB4*, białko MDR3, marskość wątroby, kwas ursodeoksycholowy, przeszczepienie wątroby, cholestasis, progressive familial intrahepatic cholestasis, *ABCB4* gene, MDR3 protein, liver cirrhosis, ursodeoxycholic acid, liver transplantation

## Abstract

Postępująca rodzinna cholestaza wewnątrzwątrobowa typu 3 (PFIC-3) należy do grupy rodzinnych cholestaz wewnątrzwątrobowych, dziedziczonych w sposób autosomalny recesywny. Patogeneza choroby wiąże się z obecnością patogennych wariantów molekularnych w genie ABCB4. Dotychczas, w literaturze opisano ok. 200 pacjentów z różnymi schorzeniami wątroby i dróg żółciowych, stanowiących ekspresję kliniczną PFIC-3.

Celem pracy jest charakterystyka patogenezy, obrazu klinicznego, diagnostyki oraz leczenia PFIC-3 na podstawie aktualnego przeglądu piśmiennictwa.

## Abstract (en)

Progressive familial intrahepatic cholestasis is caused by mutations in the ABCB4 gene and belongs to the family of familial intrahepatic cholestais disorders inherited in an autosomal recessive pattern. To date, about 200 patients with various hepatobiliary disorders associated with ABCB4 gene mutations have been described in the literature.

The aim of this manuscript was to describe the pathogenesis, clinical presentation, diagnostic process and treatment of progressive familial intrahepatic cholestais type 3, based on the literature review.

## Definicja

Postępująca rodzinna cholestaza wewnątrzwątrobowa typu 3 (ang. *Progressive Familial Intrahepatic Cholestasis type 3*, PFIC-3) [OMIM 602347], obok postępującej rodzinnej cholestazy wewnątrzwątrobowej typu 1 i 2 (PFIC-1, PFIC-2) oraz deficytu białka TJP-2 (ang. *Tight Junction Protein 2*) należy do grupy rodzinnych cholestaz wewnątrzwątrobowych, dziedziczonych w sposób autosomalny recesywny, będących przyczyną postępującego uszkodzenia wątroby [1, 2].

## Patogeneza

Patogeneza choroby wiąże się z obecnością patogennych wariantów molekularnych genu *ABCB4* [OMIM 171060], położonego na chromosomie 7q21 i kodującego białko MDR3 (ang. *Multi-Drug Resistance class III*), należące do rodziny białek ABC (ang. *ATP-binding cassette*) i podrodziny B (ABCB) [3, 4]. Ekspresja białka MDR3 jest niemal wyłącznie ograniczona do błony kanalikowej hepatocytów, gdzie odpowiada za translokację fosfolipidów (zwłaszcza fosfatydylocholiny) do światła kanalików żółciowych [3, 4]. W wyniku braku/zmniejszonej ekspresji białka MDR3 dochodzi do produkcji żółci o zmniejszonej zawartości fosfolipidów, a także zwiększonym wysyceniu cholesterolem, wywierającej toksyczny efekt w stosunku do cholangiocytów [5, 6, 7].

Obecność patogennych wariantów molekularnych w co najmniej jednym allelu genu *ABCB4* zidentyfikowano u pacjentów z różnymi schorzeniami wątroby i dróg żółciowych:

- przejściowa cholestaza niemowlęca,
- idiopatyczna przewlekła (≥6 miesięcy) cholestaza u starszych dzieci i dorosłych,
- kamica żółciowa związana z niskim stężeniem fosfolipidów w żółci (ang. *Low Phospholipid-Associated Cholelithiasis*),
- kryptogenna marskość żółciowa wątroby,
- wewnątrzwątrobowa cholestaza ciężarnych (ang. *Intrahepatic Cholestasis of Pregnancy*),
- cholestaza polekowa (indukowana np. stosowaniem środków antykoncepcyjnych) [4, 7, 9,10,11,12].

Wszystkie powyższe zaburzenia stanowią ekspresję kliniczną postępującej rodzinnej cholestazy wewnątrzwątrobowej typu 3.

## Obraz kliniczny

Szacuje się, że zaledwie 1/3 wszystkich przypadków PFIC-3 ma swój początek w okresie niemowlęcym (bardzo rzadko w okresie noworodkowym). Zwykle pierwsze objawy choroby pojawiają się w późnym dzieciństwie, a także mogą zostać zaobserwowane dopiero u dorosłych.

W zależności od wieku wystąpienia pierwszych objawów, obraz kliniczny PFIC-3 jest różny. U najmłodszych dzieci choroba przebiega zwykle w postaci cholestazy z towarzyszącym świądem skóry (czasami świąd skóry jest jedynym objawem); w badaniu przedmiotowym z odchyleń można stwierdzić hepatomegalię, rzadziej splenomegalię, natomiast w wynikach badań laboratoryjnych podwyższoną aktywność aminotransferaz i gamma-glutamylotranspeptydazy oraz podwyższone stężenie kwasów żółciowych w surowicy.

W naturalnym przebiegu PFIC-3 dochodzi do rozwoju nadciśnienia wrotnego, stąd objawy kliniczne u starszych dzieci czy dorosłych mogą wynikać z powikłań nadciśnienia wrotnego, np. krwawienia z żylaków przełyku. W badaniu przedmiotowym typowo stwierdza się wówczas splenomegalię, a w wynikach badań laboratoryjnych cechy hipersplenizmu [1, 4, 8-9].

Pacjenci z PFIC-3 mają zwiększone ryzyko rozwoju kamicy pęcherzyka żółciowego czy wewnątrzwątrobowych dróg żółciowych (stąd wielokrotnie mylnie objawy łączone są ze stwierdzaną kamicą), zwiększone ryzyko rozwoju *hepatocarcinoma* czy *cholangiocarcinoma*, a także zwiększone ryzyko wystąpienia cholestazy polekowej czy cholestazy ciężarnych [10, 11, 12, 13]. Choroba ma charakter postępujący, do rozwoju marskości wątroby dochodzi zwykle w 2. dekadzie życia.

Danych literaturowych dotyczących obrazu klinicznego PFIC-3 w populacji pediatrycznej jest stosunkowo niewiele. Do tej pory ukazały się opisy pojedynczych przypadków lub serie kilku przypadków. Jedną z pierwszych publikacji był opis 2 przypadków autorstwa de Vree i wsp. z 1998 roku. Pierwszy opis dotyczył 3-miesięcznego chłopca z nawracającymi epizodami żółtaczki cholestatycznej i świądu skóry, który trafił do ośrodka referencyjnego w wieku 3 lat. Biopsja wątroby wykonana w tym wieku wykazała obecność dokonanej marskości narządu. Z uwagi na brak odpowiedzi na leczenie UDCA, kilka miesięcy po rozpoznaniu choroby wykonano zabieg przeszczepienia wątroby. Drugi opis dotyczył 8-miesięcznego chłopca, u którego jedynym objawem choroby był świąd skóry. W wieku 3 lat wykonano biopsję wątroby, która wykazała znaczne włóknienie narządu, bez dokonanej marskości narządu. W leczeniu stosowano UDCA, jednakże z uwagi na częściową odpowiedź i progresję choroby, w wieku 9 lat wykonano zabieg przeszczepienia wątroby.

Większą kohortę pacjentów z PFIC-3 przedstawili Jacquemin i wsp. w 2001 roku na łamach *Gastroenterology*, gdzie dokonali charakterystyki fenotypu choroby w grupie 17 pacjentów [4]. Średni wiek wystąpienia pierwszych objawów wynosił 34 miesiące (zakres 1 miesiąc – 20,5 lat), pierwszymi objawami były: żółtaczka cholestatyczna (u 12 pacjentów początek w 1. roku życia), hepatomegalia, splenomegalia, świąd skóry. U 2 starszych pacjentów (odpowiednio 13,5 lat oraz 20,5 lat) pierwszym objawem było krwawienie z żylaków przełyku. Kamicę pęcherzyka żółciowego zaobserwowano u 3 pacjentów, a kamicę wewnątrzwątrobowych dróg żółciowych u 1 pacjenta. Do rozwoju niewydolności wątroby doszło u 9 pacjentów, średnio w wieku 7,5 lat (zakres 2 lata – 12,5 lat).

Z kolei, Colombo i wsp. w 2011 roku na łamach *Journal of Paediatric Gastroenterology and Nutrition* opublikowali wyniki wieloośrodkowego badania przeprowadzonego we Włoszech, w którym opisali grupę 28 dzieci z PFIC-3 [9]. Mediana wieku wystąpienia pierwszych objawów wynosiła 10 miesięcy (zakres 1 miesiąc – 17 lat), pierwszymi objawami były: żółtaczka cholestatyczna (12/28), izolowany świąd skóry (7/28), natomiast u 7 bezobjawowych pacjentów rozpoznanie PFIC-3 zostało postawione w toku diagnostyki idiopatycznego podwyższenia aktywności aminotransferaz. Średni okres obserwacji pacjentów wynosił 72 miesiące – w tym okresie do rozwoju marskości wątroby doszło u 15 pacjentów (55%), spośród nich 6 rozwinęło niewydolność narządu – 1 pacjent zmarł w wieku 5 lat, u 5 pozostałych wykonano zabieg przeszczepienia wątroby.

Podobny odsetek pacjentów z PFIC-3 w grupie pacjentów z cholestatyczną chorobą wątroby o nieustalonej etiologii, przedstawili Gordo-Gilart i wsp. w 2016 roku. W grupie 48 dzieci z przewlekłą cholestatyczną chorobą wątroby o nieznanej przyczynie, u 9 pacjentów zidentyfikowano patogenne mutacje w 1 allelu genu *ABCB4;* mediana wieku pierwszych objawów wynosiła 2 lata.

Warto podkreślić, że o diagnostyce w kierunku PFIC-3 należy także pamiętać u starszych dzieci oraz wśród dorosłych. Vitale i wsp. w 2017 roku opublikowali wyniki badania, do którego włączono 48 pacjentów, w wieku powyżej 6 lat z kryptogenną cholestazą (definiowaną jaką wzrost aktywności gamma-glutamylotranspeptydazy lub obecność świądu skóry z towarzyszącym wzrostem stężenia kwasów żółciowych w surowicy utrzymujące się co najmniej 6 miesięcy), u których przeprowadzono badania molekularne metodą NGS (sekwencjonowanie nowej generacji) w oparciu o panel 4 genów: *ATP8B1*, *ABCB11*, *ABCB4*, *TJP2*. Wśród badanej grupy zidentyfikowano 6 dorosłych pacjentów z PFIC-3, w tym jednego pacjenta z *hepatocarcinoma*.

W 2017 roku na łamach *Journal of Hepatology* ukazały się bardzo ciekawe wyniki pracy Doge i wsp. [24]. Wśród 427 pacjentów z cholestazą o nieznanej etiologii, wyodrębniono grupę z cholestazą z podwyższoną aktywnością gamma-glutamylotranspeptydazy, a w tej podgrupie zidentyfikowano 43 pacjentów z obecnością patogennej mutacji na co najmniej 1 allelu genu *ABCB4*. Co ciekawe, aż u 13 pacjentów pierwsze objawy zaobserwowano dopiero w wieku dorosłym. Wśród kobiet wystąpienie cholestazy obserwowano w związku ze stosowaniem doustnych środków antykoncepcyjnych lub w trakcie trwania ciąży. Do chwili obecnej ukazało się wiele artykułów wiążących obecność patogennych wariantów molekularnych genu *ABCB4* z wystąpieniem wewnątrzwątrobowej cholestazy ciężarnych (także geny *ABCB11*, *ATP8B1*) [28].

## Diagnostyka

Rozpoznanie PFIC-3 jest ustalane na podstawie:

1. wywiadów – rodzinne występowanie choroby, ale także poszczególnych fenotypów: cholestazy, w tym cholestazy ciężarnych czy polekowej, kamicy pęcherzyka żółciowego czy wewnątrzwątrobowych dróg żółciowych,
2. obrazu klinicznego – żółtaczka (możliwe postaci bezżółtaczkowe), hepatomegalia, rzadziej hepatosplenomegalia, świąd skóry – czasami świąd skóry jest jedynym objawem klinicznym,
3. wyników badań dodatkowych:
  - badań laboratoryjnych – hiperbilirubinemia z przewagą stężenia bilirubiny bezpośredniej (cholestaza), podwyższona aktywność gamma-glutamylotranspeptydazy (co odróżnia PFIC-3 od PFIC-1 czy PFIC-2), podwyższone stężenie kwasów żółciowych, podwyższona aktywność aminotransferaz,
  - badań obrazowych – ultrasonografia jamy brzusznej może wykazać powiększenie wątroby czy śledziony, a także obecność złogów w pęcherzyku żółciowym czy drogach żółciowych,
  - badań histopatologicznych i immunohistochemicznych – cholestazy/zastój żółci, proliferacja przewodzików żółciowych, tworzenie rozetek hepatocytów, cechy zapalenia i/lub włóknienia, badanie immunohistochemiczne z przeciwciałami p/MDR3 – wynik negatywny [1, 4, 8-9, 18].

Cechą charakterystyczną cholestazy w przebiegu PFIC-3 jest podwyższona aktywność GGTP, która wymaga różnicowania z innymi przyczynami cholestazy zewnątrzwątrobowej – atrezja dróg żółciowych, torbiele i inne wady dróg żółciowych, kamica pęcherzyka i dróg żółciowych, czy wewnątrzwątrobowej – niedobór alfa- 1-antyproteazy, zespół Alagille’a, mukowiscydoza, toksyczne i polekowe uszkodzenie wątroby. Dla cholestazy zewnątrzwątrobowej typowe jest stwierdzenie odbarwionych stolców (także możliwe w niedoborze alfa-1-antyproteazy, zespole Alagille’a, mukowiscydozie czy innych typach PFIC), brak lub mały pęcherzyk żółciowy w badaniu USG jamy brzusznej. Cholangiografia metodą rezonansu magnetycznego pozwala wykluczyć stwardniające zapalenie dróg żółciowych lub inne patologie dróg żółciowych. Prawidłowe stężenie alfa-1-antyproteazy w surowicy oraz fenotyp alfa-1-antyproteazy pozwalają wykluczyć jej deficyt. Prawidłowy wynik badania przesiewowego noworodków w kierunku mukowiscydozy (dostępny we wszystkich województwach w Polsce od czerwca 2009 roku) oraz prawidłowe stężenie chlorków w pocie (w sytuacji braku wyniku badania przesiewowego lub w sytuacjach wątpliwych) pozwalają wykluczyć mukowiscydozę. Nieobecność wady serca, kręgów motylich, prawidłowy wynik badania oftalmologicznego w lampie szczelinowej oraz nieobecność cech dysmorfii twarzy pozwalają oddalić podejrzenie od zespołu Alagille’a [19,20,21].

Boga i wsp. przedstawili opis przypadku 15-letniego chłopca ze splenomegalią, u którego w toku diagnostyki wykonano biopsję wątroby, która wykazała obecność stłuszczenia drobnokropelkowego oraz zwiększoną zawartość miedzi (>250 μg/g suchej tkanki) w bioptacie wątroby sugerując podejrzenie choroby Wilsona. Analiza molekularna nie wykazała obecności patogennych wariantów molekularnych w genie *ATP7B*, jednakże powyższy przypadek pokazuje, że PFIC3, obok pierwotnego stwardniającego zapalenia dróg żółciowych i pierwotnego żółciowego zapalenia wątroby (dawniej pierwotnej marskości żółciowej wątroby) należy dodać do grupy jednostek chorobowych przebiegających ze zwiększonym odkładaniem miedzi w wątrobie, sugerującym chorobę Wilsona [25].

Ostateczne potwierdzenie rozpoznania PFIC-3 stanowi zwykle wynik badania molekularnego. Sekwencjonowanie nowej generacji (ang. n*ext generation sequencing*; NGS) to najbardziej zaawansowana wysokoprzepustowa metoda badania genomu człowieka, która pozwala na jednoczesne genotypowanie miliardów par zasad w znacznie krótszym czasie i za niższą cenę, w stosunku do dotychczas powszechnie stosowanego w diagnostyce genetycznej klasycznego sekwencjonowania według Sangera. Może ona obejmować analizę całego genomu (ang. *Whole Genome Sequencing*; WGS), dotyczyć mapowania zmian w sekwencji kodującej wszystkich genów, stanowiącej 2% genomu, (ang. *Whole Exome Sequencing*; WES) lub obejmować analizę wybranego panelu genów (od kilkudziesięciu do kilku tysięcy). Dzięki zastosowaniu metody sekwencjonowania nowej generacji w wielu ośrodkach na świecie, ale także i w Polsce (m.in. w IP-CZD) możliwe są przesiewowe testy molekularne wykorzystujące panel kilkudziesięciu-kilkuset genów. Takie badanie może stanowić mniej kosztowną procedurę i pozwolić na postawienie rozpoznania w znacznie krótszym czasie niż opisany wyżej tok diagnostyczny [22, 23].

## Leczenie

Lekiem pierwszego rzutu jest kwas ursodeoksycholowy (UDCA) stosowany zwykle w dawce 10-20 mg/kg m.c./dobę, jednakże w ok. połowie przypadków PFIC-3 terapia UDCA jest nieskuteczna. W przypadkach z cholestazą wskazana jest suplementacja witamin rozpuszczalnych w tłuszczach (A, D, E, K). Z kolei, w przypadku rozwoju nadciśnienia wrotnego i związanych z nim powikłań stosowane jest leczenie endoskopowe (opaskowanie żylaków przełyku), a w przypadku rozwoju niewydolności wątroby pacjenci wymagają przeszczepienia narządu [1, 4, 8-9]. Co więcej, pacjenci, u których widoczna jest poprawa kliniczna w trakcie leczenia UDCA, wymagają systematycznego monitorowania z powodu zwiększonego ryzyka rozwoju kamicy żółciowej, *hepatocarcinoma*, *cholangiocarcinoma*, cholestazy polekowej czy cholestazy ciężarnych [10, 11, 12, 13].

Istnieją także pojedyncze doniesienia na temat prób zastosowania zabiegu częściowego zewnętrznego odprowadzenia żółci (ang. *Partial External Biliary Diversion*, PEBD) w grupie pacjentów z PFIC-3. Lemoine i wsp. w 2017 roku na łamach *Journal of Pediatric Surgery* przedstawili długofalową obserwację skuteczności zabiegu PEBD wśród pacjentów z PFIC, w tym u jednego pacjenta z PFIC3 [26]. Diao i wsp., w 2013 roku, przedstawili wyniki pracy oceniającej skuteczność alternatywnej do PEBD metody, a mianowicie laparoskopowej cholecystokolostomii, polegającej na połączeniu światła pęcherzyka żółciowego i okrężnicy (bez wstawki jelitowej – w odróżnieniu od częściowego wewnętrznego odprowadzenia żółci) wśród 20 pacjentów pediatrycznych z PFIC, w tym trzech z PFIC-3; wyniki jednak nie są zadowalające [27].

## Korelacja genotyp ‒ fenotyp

Wykazano, że obecność mutacji w obu allelach genu *ABCB4*, powodująca całkowity brak ekspresji białka MDR3, wiąże się z ciężkim przebiegiem PFIC-3 w postaci szybszej progresji do marskości wątroby i słabszej odpowiedzi na leczenie UDCA [15, 16, 17]. Z kolei, pacjenci z PFIC-3, heterozygotyczni pod względem mutacji w genie *ABCB4*, mają większe szanse na pozytywne rezultaty leczenia UDCA.
